# Supplementary material for: Effectiveness and Safety of Ayurvedic Medicines in Type 2 Diabetes Mellitus Management: A Systematic Review and Meta-Analysis
Source: Front Pharmacol. 2022 Jun 8;13:821810. doi: 10.3389/fphar.2022.821810 (PMC9213670; doi:10.3389/fphar.2022.821810)
Supplement: Supplementary file 1 [file DataSheet1.zip › Supplementary Material/Forest and Funnel Plots/Punica granatum/Body weight.pdf]

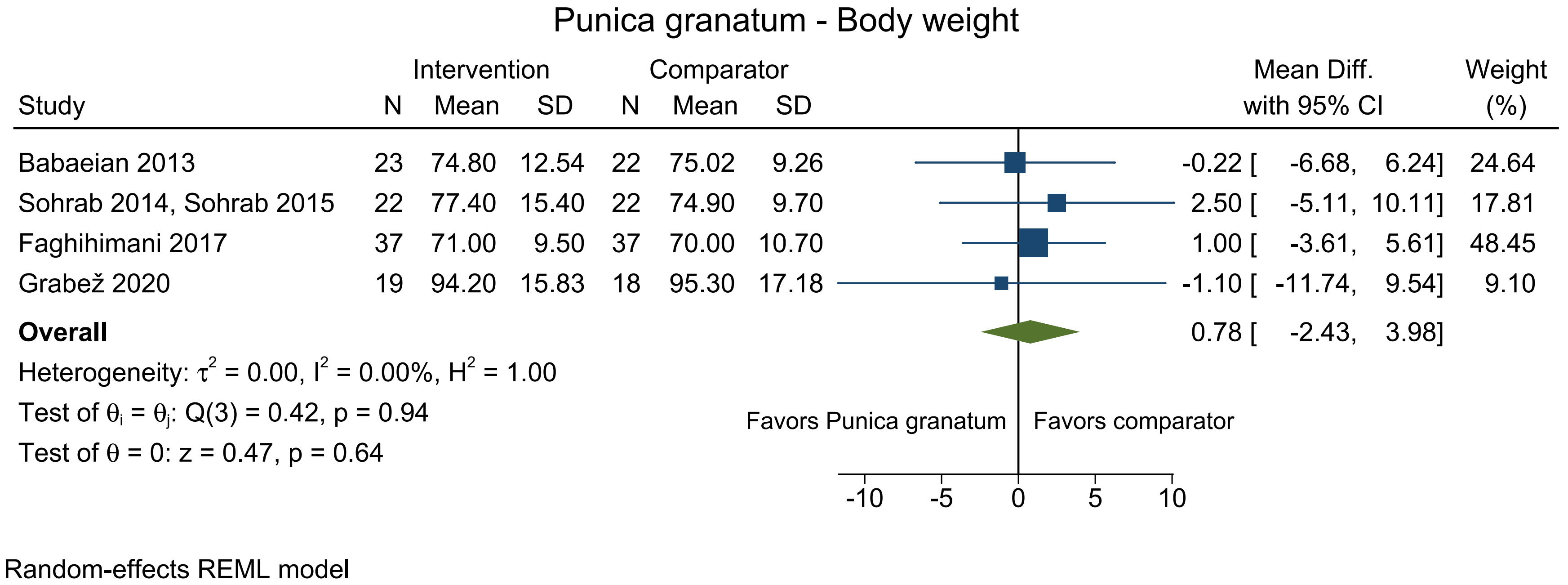
Punica granatum - Body weight
Intervention
Comparator
Study
N
Mean
SD
N
Mean
SD
Mean Diff. with 95% CI
Weight (%)
Babaeian 2013 23 74.80 12.54 22 75.02 9.26 -0.22 [-6.68, 6.24] 24.64
Sohrab 2014, Sohrab 2015 22 77.40 15.40 22 74.90 9.70 2.50 [-5.11, 10.11] 17.81
Faghihimani 2017 37 71.00 9.50 37 70.00 10.70 1.00 [-3.61, 5.61] 48.45
Grabež 2020 19 94.20 15.83 18 95.30 17.18 -1.10 [-11.74, 9.54] 9.10
Overall 0.78 [-2.43, 3.98]
Heterogeneity: $\tau^2 = 0.00$, $I^2 = 0.00\%$, $H^2 = 1.00$
Test of $\theta_i = \theta_j$: Q(3) = 0.42, p = 0.94
Test of $\theta = 0$: z = 0.47, p = 0.64
Favors Punica granatum
Favors comparator
-10
-5
0
5
10
Random-effects REML model
